# Validation and application of tissue-velocity magnetic resonance imaging for the assessment of regional diastolic velocities and diastolic performance of the right ventricle in corrected tetralogy of Fallot patients

**DOI:** 10.1186/1532-429X-13-S1-P315

**Published:** 2011-02-02

**Authors:** Anna E van der Hulst, Jos JM Westenberg, Victoria Delgado, Lucia JM Kroft, Eduard R Holman, Nico A Blom, Jeroen J Bax, Albert de Roos, Arno AW Roest

**Affiliations:** 1 Leiden University Medical Center, Leiden, Netherlands

## Objective

To compare tissue-velocity MRI (TV-MRI) and tissue Doppler imaging (TDI) to assess regional right ventricular (RV) diastolic performance at the RV free wall (RVFW) and at the RV outflow tract (RVOT) in patients with corrected Tetralogy of Fallot (cToF) and in healthy controls. To compare regional diastolic velocities and performance of the RV between cToF patients and controls. To investigate the relation between RV regional diastolic performance and RV dilatation.

## Background

The exact pathophysiological mechanism leading to RV dilatation in cToF patients with pulmonary regurgitation (PR) is not fully understood. Dysfunction of the surgically damaged RVOT may play an important role. PR causes an altered RV filling pattern during diastole and therefore, assessment of diastolic performance of the RV inlet and outlet may provide insight into the adaptive response of the RV components to chronic volume overload, ultimately leading to RV dilatation.

## Methods

Thirty-four cToF patients and 19 controls were studied. PR was assessed with three-dimensional flow assessment and RV dimensions were measured with planimetry. Early (E’) and late (A’) peak diastolic velocity and E’/A’ were assessed with TV-MRI and TDI at the RVFW and at the RVOT.

## Results

Strong correlations were observed between TV-MRI and TDI at both regions of the RV (RVFW E’: r=0.92, p<0.001, A’: r=0.92, p<0.001; RVOT E’: r=0.92, p<0.01, A’: r=0.95, p<0.001). With both techniques, E’/A’ at the RVOT was increased in cToF patients (Table 1). Regional diastolic performance at the RVOT (assessed with both VE-MRI and TDI) was significantly related to RV end-diastolic volume, even after correction for pulmonary regurgitation fraction (Table 2).

**Table 1:** Regional diastolic performance in cToF patients and controls

	cToF Patients	Controls	p-value
**RVFW**			
E’/A’			
TV-MRI	2.3 (1.8 - 2.9)	1.9 (1.7-2.6)	0.095
TDI	2.4 (2.0 - 3.1)	2.0 (1.7-2.4)	0.038
**RVOT**			
E’/A’			
TV-MRI	3.7 (2.5 - 5.8)	2.5 (1.9 - 3.3)	0.008
TDI	4.0 (2.6 -7.7)	2.9 (1.9 -3.7)	0.015

Abbreviations: RVFW: right ventricular free wall, RVOT: right ventricular outflow tract, TDI: tissue Doppler imaging, TV-MRI: velocity-encoded MRI.

**Table 2:** Relation between regional diastolic performance and RV end-diastolic volume

	Univariate				Corrected for PI			
	B	95% CI	r	p-value	B	95% CI	r	p-value

**RVFW**								
TV-MRI E''/A''	5.2	5.0 - 15.4	0.14	0.31	5.0	2.2 - 12.2	0.72	0.17
TDI E''/A''	6.9	3.8 - 17.6	0.18	0.20	6.2	1.3 - 13.7	0.72	0.10
**RVOT**								
TV-MRI E''/A''	7.1	3.4 - 10.9	0.48	<0.01	3.2	0.01-1.5	0.73	0.05
TDI E''/A''	7.6	4.7 - 10.5	0.60	<0.01	4.4	1.8 - 7.1	0.76	0.02

## Conclusions

TV-MRI and TDI show a strong correlation for the assessment of regional diastolic velocities and performance of the RV in cToF patients and in healthy controls. Regional diastolic performance of the RVOT is impaired in cToF patients as compared with controls. In addition to PR, impaired diastolic performance of the RVOT is related to RV dilatation.

